# Epigenetic Regulation of Inflammatory Responses in the Context of Physical Activity

**DOI:** 10.3390/genes12091313

**Published:** 2021-08-25

**Authors:** Maciej Tarnowski, Patrycja Kopytko, Katarzyna Piotrowska

**Affiliations:** 1 Department of Physiology, Pomeranian Medical University in Szczecin, 70-111 Szczecin, Poland; patrycja.kopytko@op.pl (P.K.); piot.kata@gmail.com (K.P.); 2 Institute of Physical Culture Sciences, University of Szczecin, 70-453 Szczecin, Poland

**Keywords:** epigenetics, miRNA, physical activity, exercise, inflammation

## Abstract

Epigenetic modifications occur in response to environmental changes and play a fundamental role in the regulation of gene expression. PA is found to elicit an inflammatory response, both from the innate and adaptive divisions of the immunological system. The inflammatory reaction is considered a vital trigger of epigenetic changes that in turn modulate inflammatory actions. The tissue responses to PA involve local and general changes. The epigenetic mechanisms involved include: DNA methylation, histone proteins modification and microRNA. All of them affect genetic expression in an inflammatory milieu in physical exercise depending on the magnitude of physiological stress experienced by the exerciser. PA may evoke acute or chronic biochemical and physiological responses and have a positive or negative immunomodulatory effect.

## 1. Introduction

Regular physical activity (PA) induces a broad variety of molecular changes on multiple levels in a multitude of target tissues. They trigger functional changes in skeletal muscle strength and performance, fat tissue metabolism and cardiovascular and respiratory adaptations, as well as bringing about immunomodulatory effects. PA is the element of highest importance for a healthy lifestyle and essentially reduces the risk for a myriad of so-called “diseases of civilization”, such as cardiovascular disease, type 2 diabetes mellitus (T2DM), neurodegenerative disorders and several types of cancer (reviewed in [1,2]).

Exercise training has strong immunoregulatory effects that involve the molecular cross-talk between the immune system and epigenetic changes in the genome. Exercise acts as a strong environmental factor affects epigenetic modifications, and these, in turn, regulate inflammatory responses in peripheral tissues (reviewed in [3]).

This review highlights the epigenetic alterations triggered by PA in an immunological context. Firstly, we will review the immune responses in PA. Finally, we will provide detailed, up-to-date examples of local and peripheral responses to PA on an epigenetic level.

## 2. Inflammation as an Environmental Factor Inducing Epigenetic Changes

It is known that epigenetic modifications occur in response to environmental changes and play a fundamental role in gene expression following environmental stimuli (nutrients, toxins, infections and hypoxia). DNA methylation, histone modifications and microRNA expression are influenced by environmental and lifestyle factors. Among them, we have inflammation and PA, and there is a very important and complex relationship between these phenomena. The inflammatory response passes through distinct acute, adaptive and chronic stages. These steps are controlled by specific cell types, cytokines and transcriptional signatures. It has been shown that histone modifiers and DNA-modifying enzymes alter chromatin landscapes to allow the binding of transcription factors, allowing the activation of gene expression or tightening the conformation to suppress gene expression [4]. There are several examples of genetic pathways and factors that are activated by or play key roles in the inflammatory response, and at the same time, they are subjected to epigenetic control. During inflammatory responses, sequential epigenetic modifications of pro- and anti-inflammatory signatures occur. These responses control a large number of genes, highlighting the intricate nature of such epigenetic regulatory processes. In the subsequent paragraphs, we will try to illustrate these interactions.

## 3. Inflammation in Exercise

Studying physiological responses in PA is complex, difficult and the results may be confusing. The complicacy stems from multiple variables in the assessment of PA (protocols used, intensity, frequency and volume of exercise, moments of data collection after exercise, number and fitness level of the sample subjects, markers investigated, tissues investigated and the type of sample used (animals or humans)). Thus, analysis of immune mechanisms is even more challenging since inflammation evoked by PA may be acute, chronic or low-grade, and the same cells and mediators are involved in the processes [5,6]. What is more, during PA and after PA, there is a cascade of events of changing character, usually with a broad spectrum of activity—from pro-inflammatory to anti-inflammatory (reviewed in [7]). Another point of confusion is that some inflammatory mediators, such as IL-6, have direct pro-inflammatory effects in some contexts and anti-inflammatory effects (usually non-direct) through activation of other molecules in others [8].

In this paragraph, we will present the immunological responses with regard to timing (acute and chronic) and location (local and peripheral/general), triggered by PA and exercise training. 

PA is considered one of the main components of a healthy lifestyle. PA, both in its acute form and in its chronic form, significantly affects the function of the immune system [9,10]. As previously mentioned, the regularity, intensity, duration and type of effort applied influence how the immune system responds to PA [11,12]. The general view is that moderate-intensity PA stimulates cellular immunity, while prolonged or high-intensity PA without appropriate rest can trigger decreased cellular immunity, increasing the probability of infection [10,11].

Very important aspects of PA are its pro-health benefits in certain pathological conditions that are related to chronic inflammation. Insulin resistance, T2DM, cardiovascular and chronic obstructive pulmonary diseases, colon and breast cancers, dementia and depression, are all connected with obesity and physical inactivity. Sedentary behavior is now also considered as a risk factor for Alzheimer’s disease (AD), vascular dementias and exercise contributes to promoting mental health and wellbeing [13]. Regular PA increases the size of different regions in brain structures, increases brain blood flow and decreases amyloid β formation in AD [14]. In rodent experiments with running, a decrease of IL-1β, TNF-α was observed. Running caused an increase in IL-10 and prevented the reduction of brain-derived neurotrophic factor (BDNF), and decreased the microglia inflammatory response (reviewed in [14]). Epigenetic changes caused by regular PA are crucial for healthy aging and longevity [15].

In general, PA is found to elicit an inflammatory response, both from the innate and adaptive divisions of the immunological system. The inflammation that starts is commonly recognized as sterile inflammation—the response is in the absence of pathogenic stimuli and is involved in the repair of internal disturbances. 

In response to physical stress, cellular damage and metabolic disturbances, the host tissues release danger or alarm signals (“alarmins”) that activate the pro-inflammatory branch of the innate immune system. Among these signals recognized by the innate immune system are danger-associated molecular patterns (DAMPs), such as pathogen-associated molecular patterns (PAMPs), high mobility group 1 (HMG1), uric acid, glucose, heat shock proteins and elevation of extracellular ATP concentration [16,17,18,19]. 

### 3.1. Acute or Chronic Exercise vs. Acute or Chronic Inflammation

The modulatory effect of PA on the immune system can have both positive and negative effects on immune function, producing a range of effects from strong immunological activation to immune system inhibition. There is a “J”-shaped curve [20] that illustrates the relationship between exercise intensity and susceptibility to infections. This model suggests that, while engaging in moderate PA may enhance immune function above sedentary levels, excessive amounts of prolonged, high-intensity exercise may impair immune function. It has been shown in epidemiological studies that the regular performance of moderate PA daily is associated with a reduction in the risk of upper respiratory tract infection (URTI) when compared with physical inactivity. On the contrary, there is a strong inverse relationship between infection and the intensity of endurance training (reviewed in [21,22]). Thus, depending on the magnitude of physiological stress experienced by the exerciser, PA may evoke acute or chronic biochemical and physiological responses and have a positive or negative immunomodulatory effect.

Acute physiological responses are associated with alterations in the homeostasis of the whole body (e.g., increase in the heart rate, the elevation of blood pressure, transient tissue redistribution of immune cells and muscle damage) [23,24]. Physical workout-induced skeletal muscle tissue injury and inflammation enhance the recirculation of immunoglobulins, pro- and anti-inflammatory cytokines, neutrophils, monocytes and macrophages, NK cells, cytotoxic T cells and immature B cells [7,25,26]. In certain conditions, a vigorous bout of high-intensity PA causes the formation of an “open window” 1–2 h post-exercise during which immune system functions are relatively suppressed since a decrease in peripheral blood lymphocytes is observed [27]. On the other hand, the partial suppression of cellular immunity that follows acute PA might represent an elevated state of immunosurveillance and migration of cells to more susceptible areas to infection after physical exercise (e.g., lungs and gut or muscles) [28].

When acute bouts of exercise are repeated, we regard the PA as exercise training during which some chronic effects may be produced. Even though immunosuppressive stress hormones and pro-inflammatory cytokines do not reach high levels over a short duration, moderate exercise bouts [29], over time, repetition or maintenance of regular PA leads to enhanced immunosurveillance and lowering inflammation and may be of particular clinical value for obese and diseased individuals. Moderate, regular exercise is now regarded as a key “drug” that helps with the regulation of the immune system and metabolism [30,31,32,33]. It was noted that the chronic effects of repeated exercise involve attenuation in the production and secretion of acute-phase proteins, especially CRP [34]; increased production and secretion of cytokines with anti-inflammatory function (especially IL-6 in skeletal muscle tissue and blood); followed by a rise in IL-1ra and IL-10 levels, suppression of TNF production [35,36,37] and improvement in the antioxidant power of cells [22,36,38]. The anti-inflammation induced by chronic physical exercise has been demonstrated to involve a differential cytokine response represented by increased circulating IL-6 levels. Adipose tissue has also been investigated in chronic protocols and has shown a similar anti-inflammatory pattern [39].

The picture of immunomodulation, particularly in professional athletes, seems to differ, and sometimes, it is called the “elite athlete paradox”. The aforementioned acute inflammatory actions, if not properly resolved, may lead to immunosuppression and long-term pathologies in chronic exercisers. When the intensity and frequency of exercise rise together with competition events; the associated physiological, metabolic and psychological stress; and when rest is not adequate, the problem of overtraining appears. Overtraining syndrome is characterized by reduced performance, fatigue and biochemical, immunological and physiological alterations, such as immune dysfunction, inflammation, oxidative stress and muscle damage [24,40,41,42,43]. What stands behind this state in overtrained athletes is a progression of the adaptive stage of trauma-induced in the skeletal muscles and joints to a non-adaptive stage of subclinical injury [44,45]. This activates circulating leucocytes and various tissues, including skeletal muscle tissue, to produce more pro-inflammatory cytokines and an excess of free radicals [44,45,46]. At the same time, an increased shift of fluid, plasma proteins and specific white blood cells (WBCs) develops from the circulation to the injured tissue [44,47]. 

### 3.2. Exercise as an Immunomodulator

When inflammation has a slow onset and persists for a long period of time, it becomes chronic. Chronic low-grade inflammation disrupts tissue homeostasis in ways that drive the progression of chronic conditions, such as diabetes, atherosclerosis, autoimmune diseases and cancer. This inflammatory state is characterized by elevated serum levels of IL-6 and tumor necrosis factor (TNF)-α, which are linked to the abovementioned medical conditions [48,49,50,51,52]. In contrast, exercise has been extensively used as a strategy to ameliorate the symptoms of the abovementioned pathologies [48,53]. Regular moderate-intensity PA is considered as long-lasting anti-inflammatory therapy or a non-pharmacological intervention strategy, supported by vast numbers of epidemiological studies, that acts to prevent medical conditions or to ameliorate their symptoms. The potent anti-inflammatory, antioxidant and immunosurveillance effects of exercise training have a summation effect over time in modulating carbohydrate metabolism, atherosclerosis and other disease processes [54,55,56,57]. Exercise training stimulates the pro-inflammatory cytokine IL-1b that is involved in multiple disease-related pathways. The release of IL-6 from the exercising muscle induces high levels of plasma IL-1 receptor antagonist (IL-1ra) during the recovery period that inhibits IL-1b signaling, thus attenuating its pro-inflammatory activity [55]. IL-6 also stimulates the release of cortisol, which is an anti-inflammatory hormone [37]. Similarly, inducing the anti-inflammatory cytokine IL-10, which antagonizes the actions of the quintessential pro-inflammatory cytokine TNF-α. Another receptor that plays an important role in strengthening the inflammatory response is Toll-like receptor 4 (TLR4). It is activated by numerous ligands, including oxidized low-density lipoproteins, and plays an important role in the detection of microbial molecular patterns and endogenous “danger signals”, such as those induced by tissue damage, and involved in obesity-induced insulin resistance and T2DM. Atherosclerosis and its expression is downregulated by PA [55]. Regular, controlled PA dampens chronic inflammation by decreasing oxidative stress and by augmenting antioxidant defenses consisting of enzymes, such as catalase, superoxide dismutase and glutathione peroxidase, and non-enzymatic antioxidants, including glutathione [55,56]

## 4. Epigenetic Changes Involved in Immune Responses

### 4.1. Local Response—Muscle Tissue

Skeletal muscle tissue is a primary physiological target of PA. Acute and regular exercise evokes a number of immune and adaptive responses in skeletal muscle that contribute to alterations in muscle function and physical fitness. This highly plastic tissue that can rapidly adapt to the energetic and mechanical demands placed on it through repeated bouts of exercise. Muscle damage is caused mostly by the eccentric stretch of myofibrils that includes deviation in calcium homeostasis and disruption of both the sarcoplasmic reticulum and myofibrillar proteins [57,58]. It is the moment when excessive amounts of reactive oxygen species (ROS) and DAMPs are produced and secreted into the extracellular environment [59]. Trauma-induced generation of ROS and danger signals initiate a series of signaling events that involve the aforementioned TLRs, NF-kappaB (NF-kB) and activator protein-1 (AP-1), which are all potent activators of inflammation [60,61,62]. The intracellular pathways involve mitogen-activated protein kinases (MAPKs), which together initiate processes important for inflammation, muscular adaptation and metabolic control within skeletal muscle [63,64]. Interestingly, the MAPK family (p38, erk 1/2, jnk and erk 5) are differentially activated depending on the exercise modality [61,65].

Further damage and the following sequence of activation events lead to an increase in inflammatory agents, such as prostaglandins, substance P and inflammatory cytokines (IL-1β, TNF-α and IL-6) produced by M1 macrophages, which promote the migration of immune cells (macrophages and neutrophils) to the damaged area [63,66,67]. The exercise-induced pro-inflammatory response and subsequent immune cell recruitment and polarization may also be required for adaptive responses. The repair and regeneration within the skeletal muscle are modulated by a pattern of signals involved in satellite cell activation, matrix remodeling, and neovascular formation [68].

### 4.2. Cross-Talk between Epigenetics, Muscle and PA

Skeletal muscles are among the tissues that show a high adaptive potential in response to changes in metabolic homeostasis. This feature is necessary to modify contractility and metabolism, which are adapted to the type of PA. Moreover, skeletal muscles are characterized by a high ability to regenerate in response to damage or trauma [69]. A key role in the regeneration of muscle cells is played by satellite cells, which are mesenchymal stem cells located between the membrane of the muscle fiber and basal membrane. In adults, satellite cells are resting, but in response to injury or increased contractile activity, they can re-enter the cell cycle, divide and then differentiate to join and regenerate muscle fibers. 

The response of skeletal muscles to PA is regulated by local and general factors. The contracting muscle is the source of many substances that are involved in the health-promoting effects of exercise. Interleukin-6 is produced locally in working skeletal muscle and may be responsible for the increase in plasma IL-6 levels during exercise. Physiological concentrations of IL-6 stimulate the appearance in the circulation of anti-inflammatory cytokines IL-1ra and IL-10 and inhibit the production of the pro-inflammatory cytokine TNFα [70]. After acute physical activity, an increase in Ly6C and macrophages recruitment is observed, which correlates with the secretion of IL-6 and Il-13 cytokines. This proves their important role in the development of skeletal muscle adaptation after acute physical exercise. In contrast, the function of muscle macrophages may be impaired in obesity, aging, as well as in a sedentary lifestyle [71]. 

Changes in proteins and mRNA patterns are observed after exercise. Furthermore, modulation of gene activity, methylation, histone patterns and miRNA composition were found in different types of PA research involving human and animal studies. Methylation of DNA, in most cases, specifically within a gene promoter region, leads to transcriptional repression by causing chromatin condensation and disruption of the interactions between DNA and transcription factors [72,73,74,75]. DNA methylation is performed by a group of enzymes known as DNA methyltransferases (DNMTs). Posttranslational modifications of histones include acetylation, methylation, phosphorylation, citrullination and ubiquitylation. They have various, often context-dependent, effects, including not only the ability to regulate the binding of effector molecules essential to DNA processes (such as transcription, repair and replication) but also the ability to regulate higher-order chromatin structure and stability [72,73,74,75,76]. The best-characterized are acetylation and deacetylation facilitated by histone acetyltransferases (HATs) and histone deacetylases (HDACs), respectively, and methylation and demethylation of histones that are achieved by histone methyltransferases (HMTs) and histone demethylases (HDMs), respectively. Small non-coding RNAs (microRNAs (miRNAs)) and long non-coding RNAs (lncRNAs) do not directly influence the chromatin architecture or DNA structure but vitally affect post-transcriptional regulation of gene expression [77]. The binding of miRNAs to the target sequence recruits the multiprotein complex called RNA induced silencing complex (RISC), which identifies, cleaves and degrades the target mRNA, resulting in its degradation or repression of protein translation [78].

#### 4.2.1. MicroRNAs in Muscles 

Many studies show that miRNAs regulate the expression of transcription factors and signaling mediators important to muscle biology, including such fundamental processes as the regulation of proliferation and differentiation during myogenesis. Moreover, abnormal miRNA expression has been observed in muscle diseases, including cardiac hypertrophy, and in skeletal muscular dystrophy [79]. miRNAs can be expressed in all tissues or have tissue specificity; those expressed in skeletal muscle and/or myocardium are called myomiRNA. miR-1, miR-133 and miR-133b, miR-206 and miR-499 are considered markers of myogenesis during muscle regeneration and contribute to the stabilization of neuromuscular junctions [80,81]. Some of them are also connected to the inflammatory response and were found in systemic inflammation (i.e., miR-133a, miR146a and miR181) [82]. Skeletal muscle has the ability to respond to both acute and chronic exercise, which is also seen in the alteration of gene expression, protein levels and miRNAs in muscle tissue (Table 1). These changes modify the functional and metabolic properties of the exercised muscle [83]. 

In a study by D’Souza et al., an analysis of the levels of miRNA in the tissue of the *vascus lateralis* muscle was performed (Table 1). The research group consisted of men who underwent severe resistance training. Muscle biopsies were performed at rest and at 2 and 4 hours after the end of training. Of the 30 miRNAs that were analyzed, miR-133a, miR-206, miR-486, miR-378b, miR-146a and miR-23a were regulated in response to exercise. Two hours after the end of training, an increase in miR-133a and miR-206 and a decrease in miR-378b expression were noted. However, after 4 hours, the expression of miR-486 and miR-146a increased, and the level of miR-23a decreased [84]. In another study, a panel of four miRNAs in skeletal muscle was checked; the models of exercise were resistance exercise (RE) and high-intensity interval training (HIIT) or moderate-intensity continuous training (MICT). There was a decreased level of miR-133a after RE and HIIT and RE, and there were also differences between training types (HIIT and RE and RE) and miRNA expression for miR-133a, miR-378 and miR-486. The researchers did not notice any statistically significant changes in miR-1 expression in any of the training variants. The in vitro target of myomiR 133a is the IGF1/Akt signaling pathway, which may possibly play a role in the regulation of muscle hypertrophy. These observations suggest that the simultaneous inclusion of high-intensity interval training and resistance training may promote a more favorable post-exercise anabolic response compared to RE alone by reducing miR-133a expression [91]. In a study by Rivas et al., the hypothesis that the dysregulation of miRNAs may contribute to the reduction of muscle plasticity that occurs with aging was tested. Two groups of men were recruited for the experiment: young (22 ± 1 years old) and older (74 ± 2 years old), who were doing acute resistance exercises. Skeletal muscle miRNA profiling showed altered expression of 17 out of 60 post-RE miRNAs tested in young males. Interestingly, there were no RE-induced changes in miRNA expression in older men. This fact may be related to impaired mRNA transcription, which indicates a reduced ability of aging muscles to adapt to exercise [85]. Another study found that the stress of moderate-intensity endurance training performed by untrained men increased muscle-expressed miRNAs, including miR-1, miR-133a and miR-133-b and miR-181a. In addition, it reduces the levels of miRNAs, which are elevated in muscle-wasting diseases, such as miR-9, miR-23a, miR-23b and miR-31. An increase in miR-1 expression was noted, as well as a decrease in miR-29b and miR-31 expression after short-term training. Rapid but transient regulation of miRNAs through physical exercise may indicate that they contribute to the positive adaptation of the body to endurance exercise [86]. McLean et al. conducted an EWAS-type study for which both men and women were recruited. Acute cycling exercise increased the expression of 13 miRNAs (miR-10a-5p, miR-30a-5p, miR-30d-5p, miR-22-3p, miR-128-1, miR-128-2, miR-378a-3p, miR-378f, miR-378a-5p, miR-378g, miR-378i, miR-422a and miR-532-5p) and decreased expression of miR-144-5p and miR-144-3p 30 minutes after exercise. It may be significant that miR-378 and miR-30 interact with peroxisome proliferator-activated receptor gamma coactivator 1-alpha (*PPARGC1A*) and peroxisome proliferator-activated receptor gamma coactivator 1-beta (*PPARGC1B*). miR-378a is located in intron 1 of the *PPARGC1B* gene, which may indicate that there is a regulatory network including *PPARGC1B*, *PGC-1* and miRNA that alter the expression of exercise-induced genes. It can be assumed that dysregulation of miRNA-378 expression may be one of the causes of insulin resistance in skeletal muscle and may also be responsible for the decreased *PGC-1α* response observed after exercise in insulin-resistant skeletal muscle [88]. Interesting results on miRNA expression in muscles were also obtained from studies with a chronic exercise model. Nielsen et al. noticed that, after a single endurance training, there was an increase in miR-1 and miR-133a, while after a 12-week endurance training regimen, a decrease was noted in all tested miRNAs: miR133a, miR-133b, miR-1 and mir-206. Interestingly, all myomiRNAs returned to their pre-workout expression levels 14 days after the end of the training program [89]. In one study, researchers correlated muscle and plasma miRNA levels in older people who performed knee extensor resistance exercises for 5 months. There was a trend towards lowering myomiRNA levels in muscle tissue, but there was an increase in the plasma after chronic resistance exercise. Moreover, it was found that the change in the miR-499 ratio in plasma and muscles was a sensitive marker of increased knee extensor strength after a 5-month training period [92]. Mueller et al. observed a decrease in miR-1 expression after 12 weeks of resistance training, accompanied by increased expression of the *IGF-1* gene. It is presumed that the target of miR-1 is *IGF-1*, which belongs to the group of genes strongly promoting muscle growth. The increase in *IGF-1* expression could have contributed to the increase in muscle mass observed in the volunteers [90]. In a study by Ogasawara et al., 800 miRNAs were screened before and after the acute and 12-week training period. The aim of the experiment was to investigate how resistance training affects miRNA expression and whether variability in muscle hypertrophy for RE training can be attributed to differential miRNA regulation in skeletal muscle. The results showed that 85 and 105 miRNAs altered their expression after acute and chronic training, respectively. The increased cross-sectional area of the biceps, quadriceps and hamstrings were also noted. These results indicate that miRNAs are involved in the adaptation of skeletal muscle to resistance training, although their exact role in this process is still not clear [87]. 

#### 4.2.2. DNA Methylation in Exercising Muscles

The global effect of exercise affects the methylation status in contracting muscle. Bajpeyi et al. divided 11 healthy men into two groups: good and poor responders based on DNA methylation status in the regulatory region of the *PGC1α* gene after acute endurance training. PGC1α is an important regulator of mitochondrial biogenesis and fat metabolism, and interestingly, it may also reduce the expression of pro-inflammatory genes in muscles. Only high responders who had decreased DNA methylation levels after exercise showed nucleosome repositioning in the *PGC1α* promoter, which correlated with an increase in *PGC1α* mRNA [93]. Barrés et al. examined DNA methylation on biopsies of the *vascus lateralis* muscle. The study was conducted in healthy men and women after acute physical exertion who lead a sedentary lifestyle on a daily basis. Genome-wide methylation was found to be reduced, and the promoter regions of key genes involved in the exercise response (i.e., *PGC-1α*, *TFAM*, *MEF2A* and *PDK4*) were also identified. These genes were hypomethylated immediately after acute training and remethylated 3 hours after the end of exercise. Promoter hypomethylation was accompanied by an increase in mRNA levels immediately or 3 hours after exercise [94]. Another study examined the effect of forced bed rest on *PPARGC1A* methylation status in muscles taken from 20 healthy men. An increase in methylation was detected in the *PPARGC1A* gene promoter, and 4 weeks after the initiation of aerobic training, participants were re-evaluated with regard to the level of methylation. Unfortunately, a downward trend in methylation status was found, but it did not reach the original value. A key finding in the present experiment was that bed rest was associated with a paradoxically increased insulin response of genes involved in the acute phase response and inflammation (IL-6 signaling and IL-10 and the ER stress pathway). Lack of PA is associated with the formation of inflammation and the formation of reactive oxygen species. Inefficient oxidation of nutrients due to decreased gene expression of the oxidative phosphorylation pathway and a low ratio of ATP production to oxygen consumption may result in increased ROS formation, leading to oxidative stress, which, in turn, can progress to chronic inflammation. This supports the idea that ROS production, ER stress and inflammation are involved in the development of insulin resistance caused by a lack of PA [95]. Mostly, studies on changes in methylation under the influence of exercise were performed to assess the level of genome-wide methylation. Seaborne et al. performed a methylation analysis on over 850,000 CpG islands. The PA of the studied men was planned as follows: one-time acute resistance exercises, three times a week for a period of 7 weeks, resistance exercises for a 7-week period and then resistance exercises three times a week for a period of 7 weeks. This exercise system allowed for the assessment of epigenetic regulation in various phases of muscle growth (hypertrophy, recovery and muscle memory). The results obtained indicate increased genome-wide hypomethylation in the last phase of planned activity compared to the first series of resistance exercises. Importantly, increased hypomethylation correlated with the increase in lean muscle mass. The following genes that were hypomethylated and showed an increase in their expression were also identified: *AXIN1*, *GRIK2*, *CAMK4* and *TRAF1* in all phases of PA. The *UBR5*, *RPL35a*, *HEG1* and *PLA2G16*, *SETD3* genes showed hypomethylation and increased expression after resistance exercises compared to baseline and interestingly showed an even greater increase in both hypomethylation and gene expression after restarting training. It is supposed that the identified genes are involved in the regulation of muscle memory [96]. Lindholm et al. conducted a 3-month study involving one-leg training in 23 healthy volunteers. Muscle biopsies were performed at rest, before and after the training period from both legs (the untrained leg was the control). Methylation changes were detected at 4919 sites in the genome of the trained leg. Of these, 839 sites showed an absolute change of at least 5% in the mean post-workout methylation level. In addition, it identified 4076 genes with altered expression that are, among others, elements of pathways involved in training adaptation, muscle training and the regulation of the body’s immune system. Hypermethylated genes were mainly associated with muscle remodeling and glucose metabolism, while hypomethylated genes were associated with inflammatory/immunological processes and transcriptional regulation. This suggests that the changes in methylation observed during training were not a random genome-wide effect but rather a controlled process that likely contributes to skeletal muscle adaptation to endurance training [97].

#### 4.2.3. Histone Modifications in Muscles after PA

Epigenetic changes due to exercise also involve histone modifications. In addition to changing methylation patterns, exercise training can also cause changes in acetylation levels. Unfortunately, this topic is still insufficiently understood, and most of the research was carried out on rodent models. One study investigated whether CaMKII, an exercise-activated kinase, causes histone hyperacetylation within the NRF-1 binding site in the *Mef2A* target gene. In T2DM, the downregulation of genes involved in glucose transport (*GLUT4* and *MEF2A*) as well as oxidative phosphorylation (*NRF-1* and its target genes) are observed. *NRF-1* has been shown to not only regulate mitochondrial oxidative genes but also to control *MEF2A*, the major transcription factor for *GLUT4*. Controlling the two pathways in two ways makes *NRF-1* a candidate to be a key gene in the design of therapeutic approaches for T2DM. In exercise-induced rats, it was found that exercise-induced CaMKII activation increased histone hyperacetylation in the region of the *Nrf-1* binding site near the *Mef2a* gene, which was associated with increased *Nrf-1* binding to the *Mef2a* gene. Increased expression of *Nrf-1* and *Mef2a* was also observed. Administration of the CaMKII inhibitor (KN93) before exercise attenuated the exercise-induced increase in *Nrf-1* and *Mef2a* expression. This study identified one of the mechanisms by which *Nrf-1* regulates *Mef2a*, a critical pathway in glucose transport [98]. McGee et al. investigated the effect of exercise on global histone modifications that mediate chromatin remodeling and transcriptional activation in human skeletal muscle in response to exercise. Muscle biopsies were taken from healthy men who cycled for 60 minutes after a 12 hour fast. Increased acetylation of H3K36 was noticed. It is a site associated with the prolongation of transcription. The regulation of class IIa HDACs, which repress histone acetylation and are involved in adaptation to exercise, was also investigated. The researchers found no evidence of class IIa proteasomal degradation of HDAC but found that HDAC4 and HDAC5 were exported from the testicle during exercise. This resulted in the suppression of their inhibitory role in regulating the transcription process. Due to exercise, the protein kinases AMPK and CaMKII were activated, which induce phosphorylation-dependent HDAC class IIa nuclear export. It can be assumed that the studied signal pathway may mediate the adaptation of skeletal muscles to physical exercise [99].

### 4.3. Peripheral Responses and Circulating Factors

Exercise also induces changes in the inflammatory environment in areas beyond the skeletal muscle. It is speculated that the general inflammatory response is triggered by cytokine release from damaged muscle tissue, and they trigger ongoing loops of activation and inhibition in the periphery. Some of the cytokines released directly from skeletal muscle have been termed “myokines” (reviewed in [100,101,102]). Myokines are cytokines or peptides synthesized and released by myocytes in muscle tissue in response to muscular contractions [103]. To date, over 600 myokines have been revealed [104]. They play important roles in the autocrine regulation of metabolism in muscles, as well as the para/endocrine regulation of other tissues and organs, including e adipose tissue, liver and brain [105]. The general inflammation that spreads is characterized by the elevation of a huge spectrum of cytokines, leucocytes, cell adhesion molecules, hormones and others. As previously discussed, the most significant changes were evidenced in TNF-a, IL-1a, IL-1β, IL-6, IL-10, IL-1ra, IL-8 and IL-15 levels together with increases in serum concentrations of CK, CRP, elastase, S100-A8, S100-S12, defensin, lysozyme, leucocyte elastase inhibitor and cathelicidin antimicrobial peptide (as reviewed in [41,42]). The immune reactions in the peripheral blood may also involve “endotoxemia,” as it is noted that strenuous exercise causing loss of barrier integrity in the gastrointestinal tract increases circulating lipopolysaccharide (LPS), a major component of the outer membrane of gram-negative bacteria and activator of pro-inflammatory TLRs [106,107].

The epigenetic changes caused by exercise are observed not only in skeletal muscle and adipose tissue but also in blood. Circulating blood gathers “information” from the whole body, and we may assume that changes in blood reflect systemic changes in response to exercise. In blood, the changes in circulating extracellular miRNA composition were noted. In addition, mononuclear cells (PBMCs) miRNAs and DNA and histone modifications were found in the peripheral blood. Many of these changes can be connected to the immune system, as PA significantly influences immune response (reviewed in [108]). 

#### 4.3.1. Circulating MicroRNAs in Exercise

There are two main sources of miRNA in blood: (i) cellular miRNAs found in PBMCs and (ii) extracellular miRNAs found in vesicles, exosomes, apoptotic bodies, high-density lipoprotein particles and protein-bound miRNAs [109]. miRNAs collected from blood during or after exercise are mainly related to skeletal muscle damage or plasticity, cardiovascular system, brain and neural tissue and the immune system [109,110]. This review, however, focuses on the inflammatory response and inflammatory modulator changes due to PA.

Circulating miRNAs connected to the immune response may function as a novel type of mediator that allows for the modulation of immune cell proliferation, maturation, activation and cytokine secretion (Table 2) [82,111]. 

miRNAs influence immune cells in patterns known from other target cells. They regulate an abundance of proteins by the inhibition of mRNA translation and enhancing mRNA degradation, but they may also directly integrate with mRNA by binding to TLR7 and TLR8 on macrophages [112,113]. The immune cells are not only target cells for miRNAs, but they also may produce them. The generation of miRNA is confirmed for T cells, B cells, neutrophils, monocytes, macrophages and NK cells [114,115,116]. The secretion of pro-inflammatory cytokines related to exercise (IL-6 and TN-Fα) is also stimulated or inhibited by different miRNAs and vice versa—the presence of these cytokines stimulates or inhibits the production of various miRNAs (Table 3) [82]. 

The miRNA response to exercise is broader, and PA varies as it is dependent on many factors, such as type of activity, duration and participants (athletes vs. non-athletes, age and gender). Available data concerning the change of miRNAs connected to the immune system and observed in blood plasma (or serum) after exercise are summarized in Table 4. 

The let-7 family is a group of miRNAs that, above other functions, are responsible for macrophage polarization and T cell differentiation (Table 1). In exercise, the expression of various members of this family was reported to increase after a marathon or systematic training with walking/running in middle-aged men and women [118,129] or decrease in acute and systematic cycling training in middle-aged participants [123,128]. miR-21 may have both pro- and anti-inflammatory properties, and its expression is reported to be rising in participants of marathons and some studies with acute cycling and sustained rowing exercise and high-intensity intervals or endurance training [117,124,125,127,130,133]. On the other hand, there was a study on acute cycling by athletes (basketball players) where the miR-21 expression was decreased [126]. Decreased miR-21 expression was also noted in a study of systematic cycling [123] and strength training [133]. Lack of change after exercise was noted in studies with single bout running and bench/leg press tests in young males [131,134]. The pro-inflammatory miR-27 was found to decrease in systematic walking training in older men and women [112]. miR-29, which is responsible for proper thymocyte function and thymus architecture [135], was found to be downregulated in middle-aged marathon runners in the study by Clauss [121], but in other studies, it was increased and dropped after 24 hours after the run [117,118]. After 24 hours, the decreased expression was also noted in a study of systemic cycling [128]. Cytokine release from monocytes can be inhibited by miR-30a [82]. The decreased expression of this miRNA was found in systemic walking/running tests performed by middle-aged participants [129]. Increased expression of miR-126 was found in marathon runners, systematic cyclists and in acute cycle tests [117,119,128]. miR-133 is connected to the skeletal muscle system, but increased expression of this miRNA was also observed in systemic inflammation [82]. Its expression after exercise was increased in treadmill tests and marathon runners [129,132,133,134]. The most studied immune system-connected miRNA is miR-146a. In different tests, the expression of this miRNA increased or decreased and was unchanged in one case (Table 3). Macrophage activation and polarization is influenced by miR-221, miR-222 and miR-223 [136,137]. Expression of these miRNAs after exercise was mainly increased. There was only one study with running, one with acute cycling, systemic walking and strength training where these miRNAs were downregulated [122,123,129,133]. In only one of these studies, blood was collected immediately after the exercise; in others, it was sometime after the end of the final bout [129]. Interestingly, there was an opposite reaction of miRNA expression due to endurance vs. strength training [133]. In endurance training, increases in miR-21, miR-146, miR-221 and miR-222 were observed, while in strength training, all tested miRNAs were downregulated [133]. The differences in expression may be explained by different test regimens (marathon, acute cycling, interval training, up or downhill walking and sustained rowing), different ages of participants and levels of training (professional athletes vs. sedentary participants or amateur athletes) and different time of blood sample collection. This last issue may appear to be very important, as blood sample collection took place immediately after the race or training or even 72 hours after training (Table 3), and some studies showed a change of expression with time, in comparison to expression detected immediately after exercise [117,123,134]. Results of tests may also be dependent on yet unknown factors (i.e., the season of the year or dietary supplementation). Ludwig and co-workers showed seasonal differences in the expression of immune-connected miR-181, miR-21, miR-29 and let-7 family members [138]. In a study of ultramarathon runners, vitamin D supplementation upregulated the expression of miR-155 and miR-223 in blood plasma after the run, in comparison to runners who did not supplement vitamin D [139]. Karvinen showed that the miR-21 expression found in vesicles changes not only in blood but also in sweat after exercise in comparison to non-exercise conditions (sauna) [127].

Not only exercise but also injuries, such as sports-related concussions, influence circulating miRNAs. A study on professional athletes revealed an increase in circulating miRNAs associated with nervous system dysfunction, including let-7a-5p, miR-223-3p and miR-26a-5p, which are also associated with immune functions [82,140]. 

Changes in the expression of circulating immune system-related miRNAs were also correlated with inflammatory and non-inflammatory markers and features. In athletes, miR-21 and miR-146 were shown to be positively correlated with height [133]. In other studies, miR-21 was correlated to adrenaline level after exercise [134] and maximal heart rate (HRmax) [122]. Halper found that the PMBC levels of miR-21 were negatively correlated to TGFβR1 in older women and positively correlated with handgrip strength in women [141]. Changes in gait speed in obese older patients were correlated with basal levels of miR-181a and miR92a [112]. After the acute cardiopulmonary training in athletes, miR-146a was correlated with the cardiac marker CKMB and inflammatory marker CRP [126]. 

The number of PBMCs rises after exercise [115,116,142], regardless of age, gender or type of PA. Different types of PBMCs also contain miRNAs that have expression changes due to PA. In neutrophils, decreased expression of miR-17, miR-20, miR-151 and let-7i was noted, while miR-181 and miR-223 were increased [115]. Natural killer (NK) cells proportionally showed the highest increase in circulation after exercise, and decreased expression was observed for miR-221 and miR-223 (opposite of the neutrophil population); however, they were increased for miR-29 [116]. In general, circulating leucocyte population changes for miR-21 and miR-126 [127] were noted. Changes in the miRNA expression in PBMCs can also be combined with changes in methylation of miRNAs locus. Changes in methylation of miR-210 and miR-21 were observed in rest after treadmill running sessions [143]. 

#### 4.3.2. Methylation Changes in PBMCs after Exercise

Exercise, or more generally PA, influences methylation status not only in muscle and adipose tissues but also in PBMCs. Generally, more sites are reported to be demethylated than hypermethylated. Methyltransferases were shown to change due to PA [144]. Methylation changes after exercise are very broad and may be dependent on the PA level or level of training of participants (athletes vs. sedentary individuals). There are studies on general PA without any special training performance. The level of PA was based on PA questionnaires or devices measuring the daily activity of participants. In studies based on PA questionnaires, methylation changes related to inflammation were found in one site in individuals declaring moderate–vigorous PA (MVPA) in their questionnaires [145]. In a study of middle-aged populations wearing single-axis accelerators, methylation of apoptosis-associated speck-like protein containing a caspase recruitment domain (ASC) (a potential marker of systemic inflammation) was found correlated with decreased levels of inflammatory cytokines (IL-6, IL-15, IL-8 and TNF-α) [146]. Light-intensity PA (LPA) was positively correlated with ASC methylation and inversely correlated to BMI [146]. In a study with bands measuring the daily activity of healthy middle-aged volunteers, the number of CpG sites whose methylation level was associated with PA was highest for basic activity, lower for MVPA and lowest for high activity [147]. DNA methylation levels were mostly decreased by PA in light and moderate/vigorous PA and immune system pathway changes were associated with LPA [147], which supports the previously shown notion that low to moderate PA is protective and immunosuppressive, but high activity without proper restitution time may lead to overtraining syndrome. 

Exercise sessions (training) are strong external stimuli that change the methylation patterns of PBMCs. After sessions of treadmill running, methylation changes were observed in pathways important not only for cardiovascular physiology but also for the immune system-connected miRs, miR-21 and miR-210 [143]. After acute aerobic exercise in untrained individuals, changes in methylation patterns in NK cells included both demethylation (57.6% of affected genes) or hypermethylation (42.4% of affected genes), with most annotations found in the FASLG gene responsible for NK cell cytotoxicity [148]. In young athletes, 72 genes in PBMCs showed different expressions due to training in comparison to sedentary controls [149]. Most genes were upregulated (mainly responsible for mitochondrial oxidative phosphorylation), and only two genes were downregulated (inflammatory response) [149]. After resistance training, changes in gene methylation in PMBCs showed enrichment in the expression of genes responsible for adhesion molecules, cancer, type 1 and type 2 diabetes, axon guidance and growth factors connected to anabolic signaling [150]. The lack of changes in methylation in the inflammatory markers *IL-6* gene and *TNF* gene due to PA have been described lately for professional cyclists [144]. Interestingly, post-exercise *TNF* gene methylation was found to be negatively correlated with BMI, exercise performance, waist circumference and mRNA expression for *TNF-α*. The authors suggest that decreases in body weight due to regular exercise may increase *TNF* DNA methylation and subsequently reduce *TNF-α* mRNA expression and chronic low-grade inflammation [144]. In this study, the mRNA for enzymes responsible for DNA methylation, DNMT3a and DNMT3b, were found downregulated after exercise, and most of the global DNA in PBMCs was found to be hypomethylated [144]. 

#### 4.3.3. Histone Modifications as a Result of PA

Epigenetic changes after exercise are also stated in histone modifications. PA is found to moderate enzymes responsible for histone acetylation/deacetylation, circulating free citrullinated histones and histone acetylation levels [151,152]. Circulating histones are cytotoxic and may trigger an inflammatory response through TLRs [151]. Extensive aerobic training causes the release of H3 histone into the bloodstream. Free circulating citrullinated H3 was correlated with the lactate level and increase of IL-10 after the bout [151]. 

The expression of enzymes responsible for histone modifications and level of acetylation seems to be dependent on body mass. The basic level of histone deacetylase 4 (HADAC4) was shown to be decreased in obese vs. lean individuals. Basal levels of acetylated histone 4 (H4ac) in LPS stimulated PBMCs from obese individuals was shown to be correlated with body mass, BMI and waist circumference [153]. In middle-aged obese individuals, 3 months of regular training (aerobic and strength) caused an increase in HADAC4 expression in PBMCs [152]. Acetylated histone 4 (H4ac) shows similar levels in PBMCs in obese vs. lean individuals before training (basic level), but LPS stimulation of PBMCs causes hyperacetylation only in cells from the obese group [153]. On the contrary, in amateur runners after a 10 km race, H4 showed no modifications in the acetylation level [154]. 

## 5. Conclusions

The studies concerning the effects of PA in different aspects of public health have great importance since PA is now considered the base of the health pyramid. Studies of epigenetic changes in tissues due to PA and in conditions of training are still in progress. The data obtained from already published research leave no doubt: daily light to moderate PA has beneficial effects in the context of inflammation and immune defense. Further studies are needed to establish the long-lasting effects and “dosages” of PA in the context of various diseases and prolongation of healthy life.

## Figures and Tables

**Table 1:** MicroRNA changes in skeletal muscle in exercise.

Type of Training	Group Size	Sex	Altered miRNAs	Reference
Resistance (acute)	9	Males	miR-24a, -133a, -146a, -206, -378b, -486	[84]
Resistance (acute)	8	Males	miR-10b-5p, -23b-3p, -24-3p, -26a-5p, -26b-5p, -27a-3p, -29a-3p, -29c-3p, -30a-5p, -30d-5p, -95-3p, -107, -126-3p, -133a, -133b, -140-3p, -181a-5p, -324-3p, -378a-5p, -423-5p, -1	[85]
Endurance (acute) +HIIT	9	Males	miR-1, -133a, -133b, -181, -9, -23a, -23b, -31	[86]
Resistance (acute + 12 weeks)	18	Males	85 miRNAs after acute training and 102 miRNAs after 12 weeks training	[87]
Cycling (acute)	6	Males + Females	miR-10a-5p, -30a-5p, -30d-5p, -22-3p, -128-1, -128-2, -378a-3p, -378f, -378a -5p, -378g, -378i, -422a, -532-5p, -144-5p, -144-3p	[88]
Endurance (acute + 12 weeks)	10	Males	mir-133a, -133b, -1, -206	[89]
Resistance	28	Males + Females	miR-1	[90]

**Table 2:** miRNAs and their functions in cells of the immune system.

miRNA	Cell Type	Function
miR-21	Macrophage Dendritic cells T cells Astrocytes extracellular	Anti/pro-inflammatory polarization Restrict T cell activation Limits Th1 and th17 polarization, increase Th17 polarization Reduced activation DAMP binding to TLR to increase inflammation
miR-27b	Macrophage	Pro-inflammatory polarization
miR-124	Macrophage Microglia T cell	Anti-inflammatory polarization, deactivation Anti-inflammatory polarization, T cell deactivation
miR-146a	Macrophage astrocyte	Reduced activation Reduced activation
miR-155	Macrophage Microglia astrocytes Dendritic cell T cell B cell	Pro-inflammatory polarization, neurotoxicity Pro-inflammatory polarization, neurotoxicity Increased activation Dendritic cell-mediated T cell activation Th1 and Th17 polarization Reduced activation and antibody production
miR-223	Macrophage Neutrophils Dendritic cell	Anti-inflammatory polarization, reduced suppressor cell activity Reduced activation Reduced activation, increased activation of Th17 cells
miR-326	T cell	Th17 differentiation
Let-7 family	Macrophage Microglia Astrocyte T cell extracellular	Anti-inflammatory polarization Dampens activation, neuroprotective Differentiation Differentiation of Th1 and Th17 cells DAMP binding to TLR to increase inflammation

**Table 3:** MicroRNAs and pro-inflammatory cytokines relations.

**TNF Alpha**			
**Is inhibited by:**	**Inhibits:**	**Is stimulated by:**	**Stimulates:**
miR-16, -26, -92, -124, -125, -130, -143, -181, -187, -193, -221, -369, -579, let-7 family	miR-23, -103, -125, -126, -128, -143, -148, -181, -221, -422	miR-21, -29, -145, -155,	miR-9, -17, -19, -27, -31, -101, -125, -146, -155, -191, -195, -221, -222, -378, -494, -513, -1280
**IL-6**			
**Is inhibited by:**	**Inhibits:**	**Is stimulated by:**	**Stimulates:**
miR-9, -23, -30, -124, -142, -146, -149, -181, -187, -199, -223, -329, let-7 family	miR-200 miR-223	miR-21, miR-29,	miR-21, -101, -181, -378

**Table 4:** Changes in the immune system connected miRNAs in various types of physical activity. IA, Immediately after; miR-133 was added to miRNAs connected to the immune system as its expression is connected to systemic inflammatory response (i.e., sepsis) [93].

Exercise Type	Participants Sex	Participants Age (Years—Mean)	Altered miRNA	Time Points for Blood Collection	
Marathon run	Males	39.1	miR-21-5p; -29-3p; -126-3p; -142-5p; -143-3p; -195-5p; -199-3p; ↓miR-103-3p	IA	[117]
			↑miR-29a-3p; let-7d-3p; let-7f-2-3p; -148a-3p; -223-3p; -223-5p ↓miR-29b-3p; -30b-5p	After 24 h	[118]
	Males	56.8	↑miR-126; -133	IA	[119]
	Males	52	↑miR-126; -133a; miR-146a	IA	[120]
	Males	40	↓miR-29b	IA	[121]
	Males	Middle-age	↓miR-30d; -181c-3p; -223-5p	15 min post-race	[122]
10 km race	Males	39	↓103a-3p	IA	[117]
	Males	Middle-age	↓miR-199b-5p; -223-3p; -223-5p	15 min post-race	[122]
Acute cycling	Males	28	↓let-7i; miR-146; -221; ↑-133a	0–3 h post exercise	[123]
	Males	19	↑miR-21; -146a; -221; -222	IA	[124]
	Males	32.4	↑miR-126	IA	[119]
	Males	20	↑miR-21; ≈miR-126; -222; -146; -155	IA	[125]
	Males	26	↓miR-21; -146a		[126]
Three repeats:	Males/females	26	↑miR-21; -222; tend to ↑miR-146	10 min after test	[127]
After sustained training	Males	19	↑miR-146a; -222	IA	[124]
After basketball season	Males	26	↑miR-221; -208b (skeletal muscle)		[126]
Systematic cycling: 12 weeks 20 weeks	Males	28	↓miR-21; let-7d; -133a; -148	3–5 d after exercise	[123]
	Males/females	43.7	↓let-7b; let-7e, miR-29c; -29b ↑miR-126; -221; -27b; -146a	24 h after last bout	[128]
Sustained rowing training	Males	19	↑miR-20a; -21; -146a; -221; -222	At rest after test	[124]
Treadmill tests: Walking (4/week, 5 months)	Males/females	69	↓27a-3p;	36–48 h after last bout	[112]
Walking/running (4/week, 8 weeks)	Males/females	30–62	↑let-7; miR-16; -26; -195; -199 ↓miR-30a; -30b; -30c; -223; -378a	IA	[129]
High intensity intervals (3/week, 8 weeks)	Females: Obese/non-obese	22/24	↑21; 150; 146a –both groups; ↑1245a,320a-non-obese; ↑223; ≈126,155,302;	48 h after last bout	[130]
Single bout running (different intensity or duration)	Males	21/22	↑miR-146a; (↑miR -133a; -222; duration dependent) ≈miR-21	IA	[131]
Uphill/downhill test	Males	27–36	↑miR-133a; -133b;	0–72 h post exercise	[132]
Acute resistance training (bench press, leg press)	Males	29.9	↓miR-146a; -221; ≈miR-21; -222; 20a	IA, post day 1 and 3	[132]
Strength training	Males	22/24	↓miR-21; -146a; -221; -222	12 h post exercise	[133]
Endurance training	Males	22/24	↑miR-21; 146a; -221; -222	12 h post exercise	[133]

↑—increase, ↓—decrease.

## References

[1] Booth F.W., Roberts C.K., Laye M.J. (2012). Lack of exercise is a major cause of chronic diseases. Compr. Physiol..

[2] Hechanova R.L., Wegler J.L., Forest C.P. (2017). Exercise: A vitally important prescription. JAAPA.

[3] Barrόn-Cambera E., Ramos-Lopez O., Gonzáles-Becerra K., Riezu-Boj J.I., Milagro F.I., Martínez -Lόpez E., Martínez J.A. (2019). Epigenetic Modifications as Outcomes of Exercise Interventions Related to Specific Metabolic Alternations: A Systematic Review. Lifestyle Genom..

[4] McCall C.E., Yoza B., Liu T., El Gazzar M. (2010). Gene-specific epigenetic regulation in serious infections with systemic inflammation. J. Innate. Immun..

[5] Rea I.M., Gibson D.S., McGilligan V., McNerlan S.E., Alexander H.D., Ross O.A. (2018). Age and age-related diseases: Role of inflammation triggers and cytokines. Front. Immunol..

[6] Medzhitov R. (2008). Origin and physiological roles of inflammation. Nature.

[7] Miles M.P., Wilson S., Yeoman C.J. (2019). Physical Activity and Inflammation Phenotype Conversion. J. Clin. Exerc. Physiol..

[8] Fischer C.P. (2006). Interleukin-6 in acute exercise and training: What is the biological relevance?. Exerc. Immunol. Rev..

[9] Pedersen B.K., Hoffman-Goetz L. (2000). Exercise and the immune system: Regulation integration and adaption. Physiol. Rev..

[10] Leandro C.G., Castro R.M., Nascimento E., Pithon-Curi T.C., Curi R. (2007). Adaptative mechanisms of the immune system in response to physical training. Rev. Bras. Med. Esporte..

[11] Laddu D.R., Lavie C.J., Phillips S.A., Arena R. (2020). Physical activity for immunity protection: Inoculating populations with healthy living medicine in preparation for the next pandemic. Prog. Cardiovasc. Dis..

[12] Simpson R.J., Katsanis E. (2020). The immunological case for staying active during the COVID-19 pandemic. Brain Behav. Immun..

[13] Rea I.M. (2017). Towards aging well: Use it or lose it: Exercise, epigenetic amd cognition. Biogerentology.

[14] De La Rosa A., Olaso-Gonzalez G., Arc-Chagnaud C., Millan F., Salvador-Pascual A., García-Lucerga C., Blasco-Lafarga C., Garcia-Domingues E., Carretero A., Correas A.G. (2020). Physical exercisein the preventionand treatment of Alzheimer’s disease. J. Sport Health Sci..

[15] Morris B.J., Wilcox B.J., Donlon T.A. (2019). Genetic And epigenetic regulation of human aging and longevity. BBA-Mol. Basis Dis..

[16] Kharraz Y., Guerra J., Mann C.J., Serrano A.L., Munoz-Canoves P. (2013). Macrophage plasticity and the role of inflammation in skeletal muscle repair. Mediat. Inflamm..

[17] Lech M., Anders H.J. (2013). Macrophages and fibrosis: How resident and infiltrating mononuclear phagocytes orchestrate all phases of tissue injury and repair. Biochim. Biophys. Acta.

[18] Maslanik T., Mahaffey L., Tannura K., Beninson L., Greenwood B.N., Fleshner M. (2013). The inflammasome and danger associated molecular patterns (DAMPs) are implicated in cytokine and chemokine responses following stressor exposure. Brain Behav. Immun..

[19] Gong T., Liu L., Jiang W., Zhou R. (2020). DAMP-sensing receptors in sterile inflammation and inflammatory diseases. Nat. Rev. Immunol..

[20] Nieman D.C. (1994). Exercise, infection and immunity. Int. J. Sports Med..

[21] Gleeson M. (2007). Immune function in sport and exercise. J. Appl. Physiol..

[22] Da Luz-Sheffer D., Latini A. (2020). Exercise-induced immune system response: Anti-inflammatory status on peripheral and central organs. Biochim. Biophys. Acta Mol. Basis Dis..

[23] Budde H., Schwarz R., Velasques B., Ribeiro P., Holzweg M., Machado S., Brazaitis M., Staack F., Wegner M. (2016). The need for differentiating between exercise, physical activity, and training. Autoimmun. Rev..

[24] Peake J.M., Neubauer O., Walsh N.P., Simpson R.J. (2017). Recovery of the immune system after exercise. J. Appl. Physiol..

[25] Bigley A.B., Rezvani K., Chew C., Sekine T., Pistillo M., Crucian B., Bollard C.M., Simpson R.J. (2014). Acute exercise preferentially redeploys NK-cells with a highly-differentiated phenotype and augments cytotoxicity against lymphoma and multiple myeloma target cells. Brain Behav. Immun..

[26] LaVoy E.C., Bollard C.M., Hanley P.J., Blaney J.W., O’Connor D.P., Bosch J.A., Simpson R.J. (2015). A single bout of dynamic exercise enhances the expansion of MAGE-A4 and PRAME-specific cytotoxic T-cells from healthy adults. Exerc. Immunol. Rev..

[27] Kakanis M.W., Peake J., Brenu E.W., Simmonds M., Gray B., Hooper S.L., Marshall-Gradisnik S.M. (2010). The open window of susceptibility to infection after acute exercise in healthy young male elite athletes. Exerc. Immunol. Rev..

[28] Campbell J.P., Turner J.E. (2018). Debunking the myth of exercise-induced immune suppression: Redefining the impact of exercise on immunological health across the lifespan. Front. Immunol..

[29] Nieman D.C., Henson D.A., Austin M.D., Brown V.A. (2005). Immune response to a 30-minute walk. Med. Sci. Sports Exerc..

[30] Adams G.R., Zaldivar F.P., Nance D.M., Kodesh E., Radom-Aizik S., Cooper D.M. (2011). Exercise and leukocyte interchange among central circulation, lung, spleen, and muscle. Brain Behav. Immun..

[31] Karstoft K., Pedersen B.K. (2016). Exercise and type 2 diabetes: Focus on metabolism and inflammation. Immunol. Cell Biol..

[32] Pedersen B.K. (2017). Anti-inflammatory effects of exercise: Role in diabetes and cardiovascular disease. Eur. J. Clin. Investig..

[33] Zouhal H., Zare-Kookandeh N., Haghighi M.M., Daraei A., de Sousa M., Soltani M., Abderrahman A.B., Tijani J.M., Hackney A.C., Laher I. Physical activity and adipokine levels in individuals with type 2 diabetes: A literature review and practical applications. Rev. Endocr. Metad. Disord..

[34] Kasapis C., Thompson P.D. (2005). The effects of physical activity on serum C-reactive protein and inflammatory markers: A systematic review. J. Am. Coll. Cardiol..

[35] Petersen A.M.W., Pedersen B.K. (2005). The anti-inflammatory effect of exercise. J. Appl. Physiol..

[36] Svensson M., Lexell J., Deierborg T. (2015). Effects of physical exercise on neuroinflammation, neuroplasticity, neurodegeneration, and behavior. Neurorehabil. Neural. Repair.

[37] Steensberg A., Fischer C.P., Keller C., Møller K., Pedersen B.K. (2003). IL-6 enhances plasma IL-1ra, IL-10, and cortisol in humans. Am. J. Physiol. Metab..

[38] Ji L.L. (1999). Antioxidants and oxidative stress in exercise. Proc. Soc. Exp. Biol. Med..

[39] Lira F.S., Rosa J.C., Yamashita A.S., Koyama C.H., Batista M.L., Seelaender M. (2009). Endurance training induces depot--specific changes in IL-10/TNF-a ratio in rat adipose tissue. Cytokine.

[40] Meeusen R., Duclos M., Gleeson M., Rietjens G., Steinacker J., Urhausen A. (2006). Prevention, diagnosis and treatment of the overtraining syndrome. Eur. J. Sports Sci..

[41] Nieman D.C., Groen A.J., Pugachev A., Vacca G. (2018). Detection of functional overreaching in endurance athletes using proteomics. Proteomes.

[42] Nieman D.C., Wentz M.L. (2019). The compelling link between physical activity and the body’s defence system. J. Sports Heath Sci..

[43] Nieman D.C. (1997). Immune response to heavy exertion. J. Appl. Physiol..

[44] Smith L.L. (2000). Cytokine hypothesis of overtraining: A physiological adaptation to excessive stress?. Med. Sci. Sports Exerc..

[45] Bassel-Duby R., Olson E.N. (2006). Signaling pathways in skeletal muscle remodeling. Annu. Rev. Biochem..

[46] Niess A.M., Dickhuth H.H., Northoff H., Fehrenbach E. (1999). Free radicals and oxidative stress in exercise–immunological aspects. Exerc. Immunol. Rev..

[47] Da Rocha A.L., Pinto A.P., Kohama E.B., Pauli J.R., de Moura L.P., Cintra D.E., Ropelle E.R., da Silva A.S.R. (2019). The proinflammatory effects of chronic excessive exercise. Cytokine.

[48] Gleeson M., Bishop N.C., Stensel D.J., Lindley M.R., Mastana S.S., Nimmo M.A. (2011). The anti-inflammatory effects of exercise: Mechanisms and implications for the prevention and treatment of disease. Nat. Rev. Immunol..

[49] Hotamisligil G.S. (2006). Inflammation and metabolic disorders. Nature.

[50] Leonard B.E. (2007). Inflammation, depression and dementia: Are they connected?. Neurochem. Res..

[51] Rook G.A., Dalgleish A. (2011). Infection, immunoregulation, and cancer. Immunol. Rev..

[52] Shoelson S.E., Lee J., Goldfine A.B. (2006). Inflammation and insulin resistance. J. Clin. Investig..

[53] Vina J., Sanchis-Gomar F., Martinez-Bello V., Gomez-Cabrera M.C. (2012). Exercise acts as a drug; the pharmacological benefits of exercise. Br. J. Pharmacol..

[54] Antunes B.M., Cayres S.U., Lira F.S., Fernandes R.A. (2016). Arterial thickness and immunometabolism: The mediating role of chronic exercise. Curr. Cardiol. Rev..

[55] Lancaster G.I., Febbraio M.A. (2014). The immunomodulating role of exercise in metabolic disease. Trends Immunol..

[56] Valacchi G., Virgili F., Cervellati C., Pecorelli A. (2018). OxInflammation: From subclinical condition to pathological biomarker. Front. Physiol..

[57] Koelwyn G.J., Wennerberg E., Demaria S., Jones L.W. (2015). Exercise in regulation of inflammation-immune axis function in cancer initiation and progression. Oncology.

[58] Niess A.M., Simon P. (2007). Response and adaptation of skeletal muscle to exercise-the role of reactive oxygen species. Front. Biosci..

[59] Proske U., Morgan D.L. (2001). Muscle damage from eccentric exercise: Mechanism, mechanical signs, adaptation and clinical applications. J. Physiol..

[60] Hollander J., Fiebig R., Gore M., Ookawara T., Ohno H., Ji L.L. (2001). Superoxide dismutase gene expression is activated by a single bout of exercise in rat skeletal muscle. Pflugers Arch. Eur. J. Physiol..

[61] Gomez-Cabrera M.C., Borras C., Pallardo F.V., Sastre J., Ji L.L., Vina J. (2005). Decreasing xanthine oxidase-mediated oxidative stress prevents useful cellular adaptations to exercise in rats. J. Physiol..

[62] Kramer H.F., Goodyear L.J. (2007). Exercise, MAPK, and NF-kappaB signaling in skeletal muscle. J. Appl. Physiol..

[63] Proske U., Allen T.J. (2005). Damage to skeletal muscle from eccentric exercise. Exerc. Sport Sci. Rev..

[64] Kim J.S., Saengsirisuwan V., Sloniger J.A., Teachey M.K., Henriksen E.J. (2006). Oxidant stress and skeletal muscle glucose transport: Roles of insulin signaling and p38 MAPK. Free Radic. Biol. Med..

[65] Widegren U., Wretman C., Lionikas A., Hedin G., Henriksson J. (2000). Influence of exercise intensity on ERK/MAP kinase signalling in human skeletal muscle. Pflug. Arch. Eur. J. Physiol..

[66] Smith L.L., Anwar A., Fragen M., Rananto C., Johnson R., Holbert D. (2000). Cytokines and cell adhesion molecules associated with high-intensity eccentric exercise. Eur. J. Appl. Physiol..

[67] Peake J., Nosaka K., Suzuki K. (2005). Characterization of inflammatory responses to eccentric exercise in humans. Exerc. Immunol. Rev..

[68] Rigamonti E., Zordan P., Sciorati C., Rovere-Querini P., Brunelli S. (2014). Macrophage plasticity in skeletal muscle repair. Biomed. Res. Int..

[69] Dumont N.A., Bentzinger C.F., Sincennes M.C., Rudnicki M.A. (2015). Satellite cells and skeletal muscle regeneration. Compr. Physiol..

[70] Wärnberg J., Cunningham K., Romeo J., Marcos A. (2010). Physical activity, exercise and low-grade systemic inflammation. Proc. Nutr. Soc..

[71] Minari A.L.A., Avila F., Oyama L.M., Thomatieli-Santos R.V. (2019). Skeletal muscles induce recruitment of Ly6C^+^ macrophage subtypes and release inflammatory cytokines 3 days after downhill exercise. Am. J. Physiol. Regul. Integr. Comp. Physiol..

[72] Rothbart S.B., Strahl B.D. (2014). Interpreting the language of histone and DNA modifcations. Biochim. Biophys. Acta.

[73] Mann M.R., Bartolomei M.S. (2002). Epigenetic reprogrammingin the mammalian embryo: Struggle of the clones. Genome Biol..

[74] Klose R.J., Bird A.P. (2006). Genomic DNA methylation: The mark and its mediators. Trends Biochem. Sci..

[75] Jurkowska R.Z., Jurkowski T.P., Jeltsch A. (2011). Structure and function of mammalian DNA methyltransferases. Chembiochem.

[76] Kouzarides T. (2007). Chromatin modifications and their function. Cell.

[77] Dozmorov M.G., Giles C.B., Koelsch K.A., Wren J.D. (2013). Systematic classification of non-coding RNAs by epigenomic similarity. BMC Bioinform..

[78] Carthew R.W., Sontheimer E.J. (2009). Origins and mechanisms of miRNAs and siRNAs. Cell.

[79] Deng Z., Wang D.Z., Ying S.Y. (2008). MicroRNA in Muscle Development and Function. Current Perspectives in microRNAs.

[80] Pegoraro V., Merico A., Angelini C. (2019). MyomiRNAs Dysregulation in ALS Rehabilitation. Brain Sci..

[81] Sempere L.F., Freemantle S., Pitha-Rowe I., Moss E., Dmitrovsky E., Ambros V. (2004). Expression profiling of mammalian microRNAs uncovers a subset of brain-expressed microRNAs with possible roles in murine and human neuronal differentiation. Genome. Biol..

[82] Benz F., Roy S., Trautwein C., Roderburg C., Luedde T. (2016). Circulating MicroRNAs as Biomarkers for Sepsis. Int. J. Mol. Sci..

[83] Hernández-Hernández J.M., García-González E.G., Brun C.E., Rudnicki M.A. (2017). The myogenic regulatory factors, determinants of muscle development, cell identity and regeneration. Semin. Cell Dev. Biol..

[84] D’Souza R.F., Markworth J.F., Aasen K.M.M., Zeng N., Cameron-Smith D., Mitchell C.J. (2017). Acute resistance exercise modulates microRNA expression profiles: Combined tissue and circulatory targeted analyses. PLoS ONE.

[85] Rivas D.A., Lessard S.J., Rice N.P., Lustgarten M.S., So K., Goodyear L.J., Parnell L.D., Fielding R.A. (2014). Diminished skeletal muscle microRNA expression with aging is associated with attenuated muscle plasticity and inhibition of IGF-1 signaling. FASEB J..

[86] Russell A.P., Lamon S., Boon H., Wada S., Güller I., Brown E.R., Chibalin A.V., Zierath J.R., Snow R.J., Wadley G.D. (2013). Regulation of miRNAs in human skeletal muscle following acute endurance exercise and short-term endurance training. J. Physiol..

[87] Ogasawara R., Akimoto T., Umeno T., Sawada S., Hamaoka T., Fujita S. (2016). MicroRNA expression profiling in skeletal muscle reveals different regulatory patterns in high and low responders to resistance training. Physiol. Genom..

[88] McLean C.S., Mielke C., Cordova J.M., Langlais P.R., Bowen B., Miranda D., Coletta D.K., Mandarino L.J. (2015). Gene and MicroRNA Expression Responses to Exercise; Relationship with Insulin Sensitivity. PLoS ONE.

[89] Nielsen S., Scheele C., Yfanti C., Akerström T., Nielsen A.R., Pedersen B.K., Laye M.J. (2010). Muscle specific microRNAs are regulated by endurance exercise in human skeletal muscle. J. Physiol..

[90] Mueller M., Breil F.A., Lurman G., Klossner S., Flück M., Billeter L., Däpp C., Hoppeler H. (2011). Different molecular and structural adaptations with eccentric and conventional strength training in elderly men and women. Gerontology.

[91] Fyfe J.J., Bishop D.J., Zacharewicz E., Russell A.P., Stepto N.K. (2016). Concurrent exercise incorporating high-intensity interval or continuous training modulates mTORC1 signaling and microRNA expression in human skeletal muscle. Am. J. Physiol. Regul. Integr. Comp. Physiol..

[92] Zhang T., Birbrair A., Wang Z.M., Messi M.L., Marsh A.P., Leng I., Niclas B.J., Delbono O. (2015). Improved knee extensor strength with resistance training associates with muscle specific miRNAs in older adults. Exp. Gerontol..

[93] Bajpeyi S., Covington J.D., Taylor E.M., Stewart L.K., Galgani J.E., Henagan T.M. (2017). Skeletal Muscle PGC1α -1 Nucleosome Position and -260 nt DNA Methylation Determine Exercise Response and Prevent Ectopic Lipid Accumulation in Men. Endocrinology.

[94] Barrès R., Yan J., Egan B., Treebak J.T., Rasmussen M., Fritz T., Caidahl K., Krook A., O’Gorman D.J., Zierath J.R. (2012). Acute exercise remodels promoter methylation in human skeletal muscle. Cell Metab..

[95] Alibegovic A.C., Sonne M.P., Højbjerre L., Bork-Jensen J., Jacobsen S., Nilsson E., Faerch K., Hiscock N., Mortensen B., Friedrichsen M. (2010). Insulin resistance induced by physical inactivity is associated with multiple transcriptional changes in skeletal muscle in young men. Am. J. Physiol. Endocrinol. Metab..

[96] Seaborne R.A., Strauss J., Cocks M., Shepherd S., O’Brien T.D., van Someren K.A., Bell P.G., Murgatroyd C., Morton J.P., Steward C.E. (2018). Human Skeletal Muscle Possesses an Epigenetic Memory of Hypertrophy. Sci. Rep..

[97] Lindholm M.E., Marabita F., Gomez-Cabrero D., Rundqvist H., Ekström T.J., Tegnér J., Sundberg C.J. (2014). An integrative analysis reveals coordinated reprogramming of the epigenome and the transcriptome in human skeletal muscle after training. Epigenetics.

[98] Joseph J.S., Ayeleso A.O., Mukwevho E. (2017). Exercise increases hyper-acetylation of histones on the Cis-element of NRF-1 binding to the Mef2a promoter: Implications on type 2 diabetes. Biochem. Biophys. Res. Commun..

[99] McGee S.L., Fairlie E., Garnham A.P., Hargreaves M. (2009). Exercise-induced histone modifications in human skeletal muscle. J. Physiol..

[100] Allen J., Sun Y., Woods J.A. (2015). Exercise and the Regulation of Inflammatory Responses. Prog. Mol. Biol. Transl. Sci..

[101] Ostrowski K., Rohde T., Zacho M., Asp S., Pedersen B.K. (1998). Evidence that interleukin-6 is produced in human skeletal muscle during prolonged running. J. Physiol..

[102] Lee J.H., Jun H.S. (2019). Role of Myokines in Regulating Skeletal Muscle Mass and Function. Front. Physiol..

[103] Pedersen B.K., Akerstrom T.C., Nielsen A.R., Fischer C.P. (2007). Role of myokines in exercise and metabolism. J. Appl. Physiol..

[104] Carson B.P. (2017). The potential role of contraction-induced myokines in the regulation of metabolic function for the prevention and treatment of type 2 diabetes. Front. Endocrinol..

[105] Gorgens S.W., Eckardt K., Jensen J., Drevon C.A., Eckel J. (2015). Exercise and regulation of adipokine and myokine production. Prog. Mol. Biol. Transl. Sci..

[106] Bosenberg A.T., Brock-Utne J.G., Gaffin S.L., Wells M.T., Blake G.T. (1988). Strenuous exercise causes systemic endotoxemia. J. Appl. Physiol..

[107] Brock-Utne J.G., Gaffin S.L., Wells M.T., Gathiram P., Sohar E., James M.F., Morrell D.F. (1988). Endotoxaemia in exhausted runners after a long-distance race. S. Afr. Med. J..

[108] Wang J., Liu S., Li G., Xiao J. (2020). Exercise Regulates the Immune System. Adv. Exp. Med. Biol..

[109] Makarova J.A., Maltseva D.V., Galatenko V.V., Abbasi A., Maximenko D.G., Grigoriev A.I., Tonevitsky A.G., Northoff H. (2014). Exercise immunology meets MiRNAs. Exerc. Immunol. Rev..

[110] Polakovičová M., Musil P., Laczo E., Hamar D., Kyselovič J. (2016). Circulating MicroRNAs as Potential Biomarkers of Exercise Response. Int. J. Mol. Sci..

[111] Mahesh G., Biswas R. (2019). MicroRNA-155: A Master Regulator of Inflammation. J. Interferon Cytokine Res..

[112] Zhang T., Brinkley T.E., Liu K., Feng X., Marsh A.P., Kritchevsky S., Zhou X., Nicklas B.J. (2017). Circulating MiRNAs as biomarkers of gait speed responses to aerobic exercise training in obese older adults. Aging.

[113] Fabbri M., Paone A., Calore F., Galli R., Gaudio E., Santhanam R., Lovat F., Fadda P., Mao C., Nuovo G.J. (2012). MicroRNAs bind to Toll-like receptors to induce prometastatic inflammatory response. Proc. Natl. Acad. Sci. USA.

[114] Jia Y., Wei Y. (2020). Modulators of MicroRNA Function in the Immune System. Int. J. Mol. Sci..

[115] Radom-Azik S., Zaldivar F., Oliver S., Galassetti P., Cooper D.M. (2010). Evidence for microRNA involvement in exercise-associated neutrophil gene expression changes. J. Appl. Physiol..

[116] Radom-Aizik S., Zaldivar F., Haddad F., Cooper D.M. (2013). Impact of brief exercise on peripheral blood NK cell gene and microRNA expression in young adults. J. Appl. Physiol..

[117] De Gonzalo-Calvo D., Dávalos A., Fernández-Sanjurjo M., Amado-Rodríguez L., Díaz-Coto S., Tomás-Zapico C., Montero A., García-González Á., Llorente-Cortés V., Heras M.E. (2018). Circulating microRNAs as emerging cardiac biomarkers responsive to acute exercise. Int. J. Cardiol..

[118] De Gonzalo-Calvo D., Dávalos A., Montero A., García-González Á., Tyshkovska I., González-Medina A., Soares S.M., Martínez-Camblor P., Casas-Agustench P., Rabadán M. (2015). Circulating inflammatory miRNA signature in response to different doses of aerobic exercise. J. Appl. Physiol..

[119] Uhlemann M., Möbius-Winkler S., Fikenzer S., Adam J., Redlich M., Möhlenkamp S., Hilberg T., Schuler G.C., Adams V. (2014). Circulating microRNA-126 increases after different forms of endurance exercise in healthy adults. Eur. J. Prev. Cardiol..

[120] Baggish A.L., Park J., Min P.K., Isaacs S., Parker B.A., Thompson P.D., Troyanos C., D’Hemecourt P., Dyer S., Thiel M. (2014). Rapid upregulation and clearance of distinct circulating microRNAs after prolonged aerobic exercise. J. Appl. Physiol..

[121] Clauss S., Wakili R., Hildebrand B., Kääb S., Hoster E., Klier I., Martens E., Hanley A., Hanssen H., Halle M. (2016). MicroRNAs as Biomarkers for Acute Atrial Remodeling in Marathon Runners (The miRathon Study--A Sub-Study of the Munich Marathon Study). PLoS ONE.

[122] Fernández-Sanjurjo M., Úbeda N., Fernández-García B., Del Valle M., Ramírez de Molina A., Crespo M.C., Martín-Hernández R., Casas-Agustench P., Martínez-Camblor P., de Gonzalo-Calvo D. (2020). Exercise dose affects the circulating microRNA profile in response to acute endurance exercise in male amateur runners. Scand J. Med. Sci. Sports.

[123] Nielsen S., Åkerström T., Rinnov A., Yfanti C., Scheele C., Pedersen B.K., Laye M.J. (2014). The miRNA plasma signature in response to acute aerobic exercise and endurance training. PLoS ONE.

[124] Baggish A.L., Hale A., Weiner R.B., Lewis G.D., Systrom D., Wang F., Wang T.J., Chan S.Y. (2011). Dynamic regulation of circulating microRNA during acute exhaustive exercise and sustained aerobic exercise training. J. Physiol..

[125] Zhou Q., Shi C., Lv Y., Zhao C., Jiao Z., Wang T. (2020). Circulating microRNAs in Response to Exercise Training in Healthy Adults. Front. Genet..

[126] Li Y., Yao M., Zhou Q., Cheng Y., Che L., Xu J., Xiao J., Shen Z., Bei Y. (2018). Dynamic Regulation of Circulating micro RNAs During Acute Exercise and Long Term Exercise Training in Basketball Athletes. Front. Physiol..

[127] Karvinen S., Sievänen T., Karppinen J.E., Hautasaari P., Bart G., Samoylenko A., Vainio S.J., Ahtiainen J.P., Laakkonen E.K., Kujala U.M. (2020). MicroRNAs in Extracellular Vesicles in Sweat Change in Response to Endurance Exercise. Front. Physiol..

[128] Barber J.L., Zellars K.N., Barringhaus K.G., Bouchard C., Spinale F.G., Sarzynski M.A. (2019). The Effects of Regular Exercise on Circulating Cardiovascular-related MicroRNAs. Sci. Rep..

[129] Kern F., Ludwig N., Backes C., Maldener E., Fehlmann T., Suleymanov A., Meese E., Hecksteden A., Keller A., Meyer T. (2019). Systematic Assessment of Blood-Borne MicroRNAs Highlights Molecular Profiles of Endurance Sport and Carbohydrate Uptake. Cells.

[130] Dimassi S., Karkeni E., Laurant P., Tabka Z., Landrier J.F., Riva C. (2018). Microparticle miRNAs as Biomarkers of Vascular Function and Inflammation Response to Aerobic Exercise in Obesity?. Obesity.

[131] Ramos A.E., Lo C., Estephan L.E., Tai Y.Y., Tang Y., Zhao J., Sugahara M., Gorcsan J., Brown M.G., Lieberman D.E. (2018). Specific circulating microRNAs display dose-dependent responses to variable intensity and duration of endurance exercise. Am. J. Physiol. Heart Circ. Physiol..

[132] Banzet S., Chennaoui N., Girard O., Racinais S., Drogou C., Chalabi H., Koulmann N. (2013). Changes of circdulating microRNAs levels with exercise modality. J. Appl. Physiol..

[133] Wardle S.L., Bailey M.E., Kilikevicius A., Malkova D., Wilson R.H., Venckunas T., Moran C.N. (2015). Plasma microRNA levels differ between endurance and strength athletes. PLoS ONE.

[134] Sawada S., Kon M., Wada S., Ushida T., Suzuki K., Akimoto T. (2013). Profiling of circulating microRNAs after a bout of acute resistance exercise in humans. PLoS ONE.

[135] Cron M.A., Guillochon É., Kusner L., Le Panse R. (2020). Role of miRNAs in Normal and Myasthenia Gravis Thymus. Front. Immunol..

[136] Kim G.D., Ng H.P., Patel N., Mahabeleshwar G.H. (2019). Kruppel-like factor 6 and miR-223 signaling axis regulates macrophage-mediated inflammation. FASEB J..

[137] Seeley J.J., Baker R.G., Mohamed G., Bruns T., Hayden M.S., Deshmukh S.D., Freedberg D.E., Ghosh S. (2018). Induction of innate immune memory via microRNA targeting of chromatin remodelling factors. Nature.

[138] Ludwig N., Hecksteden A., Kahraman M., Fehlmann T., Laufer T., Kern F., Meyer T., Meese E., Keller A., Backes C. (2019). Spring is in the air: Seasonal profiles indicate vernal change of miRNA activity. RNA Biol..

[139] Pastuszak-Lewandoska D., Domańska-Senderowska D., Kiszałkiewicz J., Szmigielska P., Snochowska A., Ratkowski W., Spieszny M., Klocek T., Godlewski P., Cięszczyk P. (2020). Expression levels of selected cytokines and microRNAs in response to vitamin D supplementation in ultra-marathon runners. Eur. J. Sport Sci..

[140] Svingos A.M., Asken B.M., Bauer R.M., DeKosky S.T., Hromas G.A., Jaffee M.S., Hayes R.L., Clugston J.R. (2019). Exploratory study of sport-related concussion effects on peripheral micro-RNA expression. Brain Inj..

[141] Halper B., Hofmann M., Oesen S., Franzke B., Stuparits P., Vidotto C., Tschan H., Bachl N., Strasser E.M., Quittan M. (2015). Influence of age and physical fitness on miRNA-21, TGF-β and its receptors in leukocytes of healthy women. Exerc. Immunol. Rev..

[142] Radom-Aizik S., Zaldivar F., Leu S.Y., Cooper D.M. (2009). A brief bout of exercise alters gene expression and distinct gene pathways in peripheral blood mononuclear cells of early- and late-pubertal females. J. Appl. Physiol..

[143] Denham J., O’Brien B.J., Marques F.Z., Charchar F.J. (2015). Changes in the leukocyte methylome and its effect on cardiovascular-related genes after exercise. J. Appl. Physiol..

[144] Hunter D.J., James L., Hussey B., Wadley A.J., Lindley M.R., Mastana S.S. (2019). Impact of aerobic exercise and fatty acids supplementation on global and gene-specific DNA methylation. Epigenetic.

[145] Fernández-Sanlés A., Sayols-Baixeras S., Castro de Moura M., Esteller M., Subirana I., Torres-Cuevas S., Pérez-Fernández S., Aslibekyan S., Jaume Marrugat J., Elosua R. (2020). Physical Activity and Genome-wide DNA Methylation: The REGICOR Study. Med. Sci. Sports Exerc..

[146] Nishida Y., Hara M., Higaki Y., Taguchi N., Nakamura K., Nanri H., Horita M., Shimanoe C., Yasukata J., Myoshi J. (2019). Habitual Light-intensity Physical Activity and ASC Methylation in a Middle-aged Population. Int. J. Sport Med..

[147] Caspers M., Blocquiaux S., Charlier R., Knaeps S., Lefevre J., De Bock K., Thomis M. (2018). Intensity-Specific Differential Leukocyte DNA Methylation in Physical (In)Activity: An Exploratory Approach. Twin Res. Hum. Genet..

[148] Schenk A., Koliamitra C., Bauer C.J., Schier R., Schweiger M.R., Bloch W., Zimmer P. (2019). Impact of Acute Aerobic Exercise on Genome-Wide DNA-Methylation in Natural Killer Cells-A Pilot Study. Genes.

[149] Liu D., Wang R., Grant A.R., Zhang J., Gordon P.M., Wei Y., Chen P. (2017). Immune adaptation to chronic intense exercise training: New microarray evidence. BMC Genomics.

[150] Denham J., Marques F.Z., Bruns E.L., O’Brien B.J., Charchar F.J. (2016). Epigenetic changes in leukocytes after 8 weeks of resistance exercise training. Eur. J. Appl. Physiol..

[151] Stawski R., Walczak K., Perdas E., Prymont-Przymińska A., Zwolińska A., Kosielski P., Budlewski T., Padula G., Jerczynska H., Nowak D. (2021). Increased Circulating H3 Histone in Response to Repeated Bouts of Exercise Does Not Associate with Parallel Alterations of Cell-Free DNA. Biology.

[152] Abu-Farha M., Tiss A., Abubaker J., Khadir A., Al-Ghimlas F., Al-Khairi I., Baturcam E., Cherian P., Elkum N., Hammad M. (2013). Proteomics analysis of human obesity reveals the epigenetic factor HDAC4 as a potential target for obesity. PLoS ONE.

[153] Dorneles G.P., Boeira M.C.R., Schipper L.L., Silva I.R.V., Elsner V.R., Dal Lago P., Peres A., Romão P.R.T. (2017). Acute Strenuous Exercise Induces an Imbalance on Histone H4 Acetylation/Histone Deacetylase 2 and Increases the Proinflammatory Profile of PBMC of Obese Individuals. Oxid. Med. Cell Longev..

[154] De Silveira F.P., Basso C., Raupp W., Dalipaz M., Bertoldi K., Rodrigues-Siquiera I., Lago P.D., de Souza M.P., Elsner V.R. (2017). BDNF levels are increased in peripherial blood of middle aged amateur runners with no changes on Histone H4 acetylation levels. J. Physiol. Sci..

